# Effect of pulsed intravenous methylprednisolone with alternative low-dose prednisone on high-risk IgA nephropathy: a 18-month prospective clinical trial

**DOI:** 10.1038/s41598-021-03691-0

**Published:** 2022-01-07

**Authors:** Yan Li, Rongguo Fu, Jie Gao, Li Wang, Zhaoyang Duan, Lifang Tian, Heng Ge, Xiaotao Ma, Yuzhan Zhang, Ke Li, Peihao Xu, Xuefei Tian, Zhao Chen

**Affiliations:** 1 Department of Nephrology, The Second Affiliated Hospital of Xi’an Jiaotong University, 157 West Fifth Road, Xi’an, 710004 Shaanxi China; 2 School of Medicine, Royal College of Surgeons in Ireland, 123 St Stephen’s Green, Dublin 2, Ireland; 3 Section of Nephrology, Department of Internal Medicine, Yale University School of Medicine, New Haven, CT 06520 USA

**Keywords:** Nephrology, IgA nephropathy

## Abstract

Full-dose prednisone (FP) regimen in the treatment of high-risk immunoglobulin A nephropathy (IgAN) patients, is still controversial. The pulsed intravenous methylprednisolone combined with alternative low-dose prednisone (MCALP) might have a more favorable safety profile, which has not been fully investigated. Eighty-seven biopsy-proven IgAN adult patients and proteinuria between 1 and 3.5 g/24 h after ACEI/ARB for at least 90 days were randomly assigned to 6-month therapy: (1) MCALP group: 0.5 g of methylprednisolone intravenously for three consecutive days at the beginning of the course and 3rd month respectively, oral prednisone at a dose of 15 mg every other day for 6 months. (2) FP group: 0.8–1.0 mg/kg/days of prednisone (maximum 70 mg/day) for 2 months, then tapered by 5 mg every 10 days for the next 4 months. All patients were followed up for another 12 months. The primary outcome was complete remission (CR) of proteinuria at 12 months. The percentage of CR at 12th and 18th month were similar in the MCALP and FP groups (51% vs 58%, *P* = 0.490, at 12th month; 60% vs 56%, *P* = 0.714, at 18th month). The cumulative dosages of glucocorticoid were less in the MCALP group than FP group (4.31 ± 0.26 g vs 7.34 ± 1.21 g, *P* < 0.001). The analysis of the correlation between kidney biopsy Oxford MEST-C scores with clinical outcomes indicated the percentages of total remission was similar between two groups with or without M1, E1, S1, T1/T2, and C1/C2. More patients in the FP group presented infections (8% in MCALP vs 21% in FP), weight gain (4% in MCALP vs 19% in FP) and Cushing syndrome (3% in MCALP vs 18% in FP). These data indicated that MCALP maybe one of the choices for IgAN patients with a high risk for progression into ESKD.

**Trial registration:** The study approved by the Chinese Clinical Trial Registry (registration date 13/01/2018, approval number ChiCTR1800014442, https://www.chictr.org.cn/).

## Introduction

Immunoglobulin A nephropathy (IgAN), also known as Berger's disease^1^, is one of the most prevalent types of primary glomerulonephritis in the Asia–Pacific region that is an important cause of end-stage kidney disease (ESKD). IgAN occurs at any age, especially in those people at 16–35 years old, and the ratio of male patients is higher than that of female patients^2^. Recurrent gross or microscopic hematuria, varying proteinuria levels, hypertension, and progressive decline of kidney function^3^ are usually present in patients of IgAN.

Although the pathogenesis of IgAN has been not yet fully understood, Burgeoning evidence has been furnished that mucosal Infections including tonsil infection and intestinal infection play a key role in the disease progression of IgAN, genetic, environmental factors and complement may be involved in the pathogenesis as well^4^. The inflammation in glomeruli caused by an aberrantly galactose deficient IgA1-mediated immune complex deposition is thought to play an essential role in the development and progression of IgAN.

The clinical manifestations and kidney pathological findings of IgAN vary individually, which leads to different approach choices and outcomes^5^. Most affected IgAN patients develop chronic and slow-progressing kidney injury, in which approximately 30% of them will eventually progress to ESKD^6^. Persistent and massive proteinuria, especially proteinuria > 1 g/day^7,8^, and hypertension^9^ were identified as the most widely recognized two risk factors to predict renal function decline. To decrease the proteinuria levels in IgAN patients is generally accepted to be associated with slower renal functions decline and lower ESKD risk^7^. The Kidney Disease: Improving Global Outcomes (KDIGO) 2021 clinical practice guideline for the management of glomerular diseases (https://kdigo.org/guidelines/gd/) recommend to considering glucocorticoids utilization for the high-risk IgA patients when the non-immunosuppressive antiproteinuric therapies fail to reduce the proteinuria levels to below 1 g/24 h, along with the sulfonamides for prevention of the potential opportunistic infection caused by the glucocorticoids^10,11^.

As the more and more new insight into the understanding of mucosal immune system dysfunction in the pathogenesis of IgAN, and the evidence-based recommendations from the KDIGO, many clinical trials for the treatment of persistent proteinuria high-risk IgAN patients with glucocorticoids have been investigated, such as TESTING study (Therapeutic Evaluation of Steroids in IgA Nephropathy Global)^12^ and NEFIGAN study^13^, etc. The results of a prospective TESTING study^12^, in which the 95% IgAN patients were Chinese population, showed that methylprednisolone (0.6–0.8 mg/kg/day initially; maximum, 48 mg/day, then tapered the dosage at 8 mg/month) significantly reduced proteinuria levels and mitigated the progress of kidney function deterioration. Unfortunately, the TESTING study was stopped in the middle trial phase at 2.1 years than previously planned 5 years of study due to the severe adverse events of infections caused by methylprednisolone at this dosage. The results of a prospective NEFIGAN study^13^ indicated that a novel targeted-release formation of budesonide, combined with optimized renin-angiotensin system (RAS) blockade treatment, could effectively reduce proteinuria and preserve the estimated glomerular filtration rate (eGFR) in IgA patients with persistent proteinuria during the 12-month observational phase. A study reported by Pozzi et al.^14–16^ indicated that treatment with intravenous methylprednisolone (1 g methylprednisolone intravenously for 3 days at 1st, 3rd, 5th month and oral prednisone 0.5 mg/kg/day) reduced proteinuria, improved kidney function, and fewer severe adverse effects for IgAN patients whose proteinuria 1–3.5 g/24 h. A retrospective study from Ivo Laranjinha et al.^17^, comparing the efficacy of steroid pulse therapy and full-dose prednisone therapy for IgAN patients with proteinuria greater than 1 g/24 h found that steroid pulse therapy reduced the risk of relapse. Another retrospective study designed by Hotta et al.^18^ using steroid pulse therapy (500 mg/day for 3 days for three courses followed by oral prednisolone at an initial dose of 0.6 mg/kg on alternate days, with a gradual decrease in dosage over 1 year), which included IgAN patients with proteinuria greater than 0.5 g/24 h, confirmed that steroid pulse therapy had a higher response rate. Glucocorticoids treatment has been effective for IgAN patients, especially those whose pathology shows crescents, endothelial proliferation and capsular synechia etc^19^. However, the results of prospective STOP-IgA^20,21^ showed that glucocorticoids treatment has no additional favorable effects on the outcomes but more side events for the high-risk IgAN patients compared to the intensive supportive care alone during the 3-year study phase, and subsequently the extended follow-up phase (median follow-up was 7.4 years).

In spite of these findings, how to treat high-risk IgAN patients with appropriate glucocorticoid dosages and regimens effectively and safely needs more in-depth investigation. How to assess the correlation between the kidney pathological Oxford MEST-C scores and clinical outcomes for high-risk IgAN patients that have not been studied in these aforementioned clinical trials including the TESTING, STOP-IgAN et al. Based on these findings and our long-term clinical experience on the treatment of IgAN patients, we compared the efficacy and safety of pulsed intravenous methylprednisolone combined with alternative low-dose prednisone (MCALP) and full-dose prednisone (FP) regimen in high-risk IgAN patients with persistent proteinuria more than 1 g/24 h, the correlation between the kidney pathological Oxford MEST-C scores and clinical outcomes were also investigated in this study. The study was approved by the Chinese Clinical Trial Registry (registration date 13/01/2018, approval number ChiCTR1800014442, https://www.chictr.org.cn/).

## Results

### Baseline characteristics of patients

In total, 87 renal biopsy-proven IgAN patients from February 5, 2018 to January 20, 2020 were randomly assigned to the MCALP group (n = 45) and FP group (n = 42). The 44 patients (51%) were males and 43 patients (49%) were females with an average age of 35 years old. The baseline demographics, clinical characteristics, and pathologic features of patients were shown in Table 1. The baseline proteinuria levels (2.00 ± 0.75 vs 1.99 ± 0.77 g/24 h, *P* = 0.919) and eGFR-EPI (106.34 ± 19.05 vs 99.24 ± 27.09 ml/min/1.73 m^2^, *P* = 0.164) were comparable between the MCALP and FP groups. The cumulative dosages of glucocorticoid were significantly less in the MCALP group than in FP group (4.31 ± 0.26 g vs 7.34 ± 1.21 g, *P* < 0.001), respectively.

**Table 1:** Baseline demographics, clinical characteristics, and pathologic features of patients in two groups.

Variables	MCALP group (N = 45)	FP group (N = 42)	*P* value
**Clinical**			
Age, years	35 [31–39]	36 [31–41]	0.842
Sex ratio, male:female	25:20	19:23	0.336
Weight, kg	70 [58–78]	62.5 [58–78]	0.690
BMI, kg/m^2^	23.12 ± 2.92	22.94 ± 2.16	0.750
Diabetes, n (%)	0 (0)	0 (0)	–
Hypertension, n (%)	13 (28)	11 (26)	0.778
Systolic BP, mmHg	120 [111–139]	120 [110–131]	0.420
Diastolic BP, mmHg	80 [73–92]	80 [70–85]	0.164
Proteinuria, g/24 h	2.00 ± 0.75	1.99 ± 0.77	0.919
Serum BUN, mmol/L	5.11 ± 1.59	4.91 ± 1.28	0.513
Serum Cr, μmol/L	65.02 [54.45–78.33]	73.65 [58.45–84.17]	0.200
eGFR-EPI, ml/min/1.73 m^2^	106.34 ± 19.05	99.24 ± 27.09	0.164
Total serum protein, g/L	65.50 ± 7.48	65.68 ± 9.27	0.921
Serum albumin, g/L	39.09 ± 6.33	39.02 ± 6.79	0.561
Total cholesterol, mmol/L	4.58 ± 0.92	4.70 ± 1.20	0.603
Triglyceride, mmol/L	1.70 [1.19–2.13]	1.52 [1.00–2.19]	0.327
Low density lipoprotein, mmol/L	2.80 ± 0.82	3.06 ± 1.05	0.452
Fasting blood glucose, mmol/L	4.74 ± 0.57	4.79 ± 0.51	0.550
Blood uric acid, μmol/L	359.44 ± 91.21	349.86 ± 75.34	0.858
Total dosage of glucocorticoid, g	4.31 ± 0.26	7.34 ± 1.21	0.000
Treatment with ARB drugs, n (%)	44 (98)	40 (95)	0.517
Treatment with ACEI drugs, n (%)	1 (2)	2 (5)	0.517
Maximum dose of ACEI/ARB drugs, n (%)	12 (27)	10 (24)	0.759
Treatment with Chinese medicines, n (%)	31 (69)	25 (60)	0.362
**Pathologic findings (MEST-C)**			
M1, n (%)	31 (69)	26 (62)	0.493
E1, n (%)	12 (27)	6 (14)	0.154
S1, n (%)	36 (80)	29 (69)	0.240
T1/T2, n (%)	4(8)/2 (4)	3 (7)/0 (0)	0.360
C1/C2, n (%)	6 (13)/0 (0)	3 (7)/1 (2)	0.384

Values for categorical variables are given as count (percentage); values for continuous variables, as mean ± standard deviation or median [IQR]. BMI, body mass index; BP, blood pressure; eGFR-EPI, estimated glomerular filtration rate-epidemiology collaboration equation; IQR, interquartile range.

### Proteinuria and serum albumin changes in follow-up

**Figure 1:**
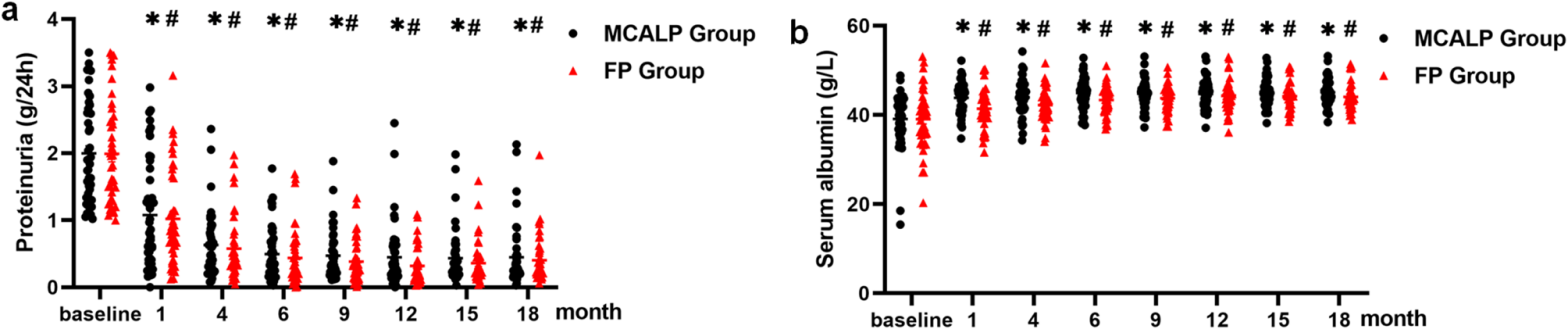
Proteinuria and serum albumin changes in follow-up. (**a**) Comparison of proteinuria. (**b**) Comparison of serum albumin. Data is shown at baseline, 1st, 4th, 6th, 9th, 12th, 15th, and 18th month of follow-up. * or ^#^*P* < 0.05 each follow-up *vs* baseline respectively.

During the observational phase of 18 months, proteinuria was decreased significantly in the MCALP group and FP group, which was shown in Fig. 1a. At the primary end-point (12th month) and the end of the trial (18th month), there was no significant difference in the reduction of proteinuria from baseline between the MCALP group and the FP group ((− 1.55 [− 1.76 to − 1.34] vs − 1.64 [− 1.89 to − 1.38] g/24 h, at 12th month, *P* = 0.139) (− 1.55 [− 1.77 to − 1.33] vs − 1.55 [− 1.83 to − 1.28] g/24 h, at 18th month, *P* = 0.604)). Meanwhile, there was no difference in the reduction of proteinuria between the two groups at baseline, 1st, 4th, 6th, 9th, and 15th month. There was a similarly significant increase in serum albumin levels in the MCALP group and the FP group at the 6th, 12th, and 18th month after treatment ((44.95 ± 3.45 vs 43.31 ± 3.24 g/L at 6th month, *P* = 0.030) (44.64 ± 3.46 vs 44.35 ± 3.66 g/L at 12th month,* P* = 0.657) (45.00 ± 3.08 vs 44.02 ± 2.99 g/L at 18th month, *P* = 0.120)) (Fig. 1b). These findings indicated that pulsed intravenous methylprednisolone combined with alternative low-dose prednisone and full-dose prednisone had a similar efficacy on the reduction of proteinuria levels and improvement of serum albumin levels in high-risk IgAN patients during the 18-month observational phase.

### Clinical remission changes in follow-up

**Figure 2:**
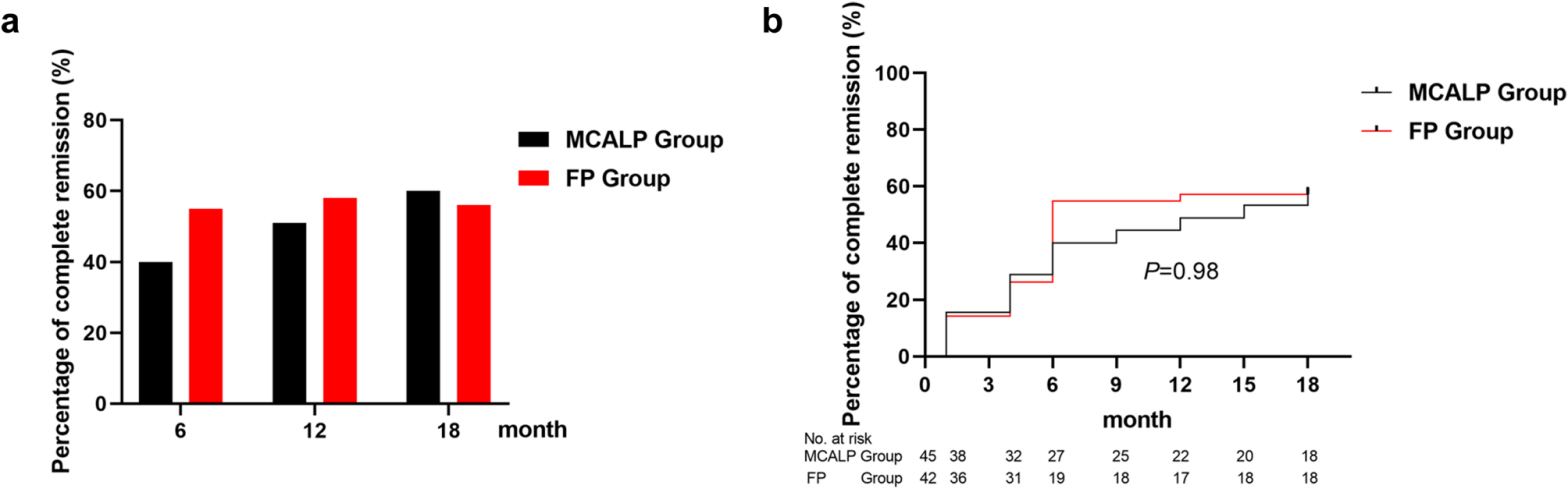
Clinical remission changes in follow-up. (**a**) The rate of complete remission at 6th, 12th, and 18th month after treatment. (**b**) Kaplan–Meier analysis for the complete remission.

At 6th month after treatment, the percentages of the patients achieving the total remission in the MCALP group (91% [83% to 100%], 41 of 45 patients) and in the FP group (90% [81% to 100%], 38 of 42 patients) were similar (*P* = 0.918) (Table 2, Fig. 2a). There was no significant difference on the complete remission rate between the MCALP group (40% [25% to 55%], 18 of 45 patients) and the FP group (55% [39% to 71%], 23 of 42 patients) (*P* = 0.168) (Table 2). During the 7–18th month follow-up, one patient in the MCALP group and two patients in the FP group who were no response to treatment at the 6th month achieved remission within 12-month follow-up (*P* = 0.503). Similarly, nine patients with partial remission in the MCALP group and four patients in the FP group with partial remission at the 6th month achieved complete remission within 12-month follow-up (*P* = 0.185).

**Table 2:** The percentage of partial remission, complete remission and total remission at the 6th, 12th, and 18th month after treatment between two groups.

Variables	MCALP group		FP group		*P* value
	n/N	Percentage (95%CI)	n/N	Percentage (95%CI)	
**Month 6**					
Partial remission	23/45	51% (36% to 66%)	15/42	36% (21% to 51%)	0.148
Complete remission	18/45	40% (25% to 55%)	23/42	55% (39% to 71%)	0.168
Total remission (proteinuria reduction ≥ 50% from baseline )	41/45	91% (83% to 100%)	38/42	90% (81% to 100%)	0.918
**Month 12**					
Partial remission	18/45	40% (25% to 55%)	13/41	32% (17% to 47%)	0.424
Complete remission	23/45	51% (36% to 66%)	24/41	58% (43% to 74%)	0.490
Total remission (proteinuria reduction ≥ 50% from baseline )	41/45	91% (83% to 100%)	37/41	90%(81% to 100%)	0.890
**Month 18**					
Partial remission	14/45	31% (17% to 45%)	14/41	34% (19% to 49%)	0.764
Complete remission	27/45	60% (45% to 75%)	23/41	56% (40% to 72%)	0.714
Total remission (proteinuria reduction ≥ 50% from baseline )	41/45	91% (83% to 100%)	37/41	90% (81% to 100%)	0.890

CI, confidence interval. n/N means event numbers/total numbers.

At 12th month, the percentages of the patients achieving the total remission in the MCALP group (91% [83% to 100%], 41 of 45 patients) and in the FP group (90% [81% to 100%], 37 of 41 patients) were similar (*P* = 0.890) (Table 2). There was no significant difference on the complete remission rate between the MCALP group (51% [36% to 66%], 23 of 45 patients) and the FP group (58% [43% to 74%], 24 of 41 patients) (*P* = 0.490) (Table 2, Fig. 2a).

At 18th month, the percentages of the patients achieving the total remission in the MCALP group (91% [83% to 100%], 41 of 45 patients) and in the FP group were also similar 90% [81% to 100%], 37 of 41 patients) (*P* = 0.890) (Table 2). (two patients in the FP group who was no response to treatment at the 6th month achieved remission gradually within 12 months follow-up and one patient who responded at 6 months turned into a no response state at 18th month). There was no significant difference on the complete remission rate between the MCALP group (60% [45% to 75%], 27 of 45 patients) and the FP group (56% [40% to 72%],23 of 41 patients) (*P* = 0.714) (Table 2, Fig. 2a).

Complete remission rates at each follow-up point between the two groups showed no significant difference during the trial (Supplementary Table 1). The average time reaching complete remission showed no significant difference between the MCALP (11.4 months [9.3 to 13.4]) and FP groups (10.3 months [8.2 to 12.4]), (*P* = 0.980) (Fig. 2).

### Relapse in follow-up

During the 7-18th month follow-up, two patients in the MCALP group and two patients in the FP group who were in response to treatment at 6th month relapsed within 12-month follow-up (*P* = 0.924).

### The correlation between the MEST-C scores for kidney pathological findings with clinical remission changes in follow-up

Next, we assessed the correlation between the kidney pathological lesions scored by the Oxford classification of MEST-C and clinical remission in the MCALP and FP groups. The results showed that the partial remission percentage in the subgroup with M1 was greater than the subgroup without M1 in the MCALP group at the 18th month (42% vs 7%, *P* = 0.020) (Supplementary Table 2). As was shown in Supplementary Table 3, the partial remission percentage in the subgroup without E1 was greater than the subgroup with E1 in the FP group at the 6th month (61% vs 17%, *P* = 0.043). In the MCALP group, the partial remission percentage in the subgroup with E1 was greater than the subgroup without E1 at 12th month (75% vs 27%, *P* = 0.004) and at 18th month (67% vs 18%, *P* = 0.002), while the complete remission percentage in the subgroup without E1 was greater than the subgroup with E1 at 12th month (61% vs 25%, *P* = 0.035) and 18th month (70% vs 33%, *P* = 0.028) (Supplementary Table 3). At 18th month, the complete remission percentage in the subgroup without S1 was greater than the subgroup with S1 in the MACLP group (89% vs 53%, *P* = 0.048) (Supplementary Table 4). The complete remission percentage in the subgroup without T1/T2 in the MCALP group was greater than the subgroup with T1/T2 at 6th month (46% vs 0%, *P* = 0.032) and continue to the 18th month (67% vs 17%, *P* = 0.020) (Supplementary Table 5); meanwhile, the total remission percentage in the subgroup without T1/T2 was greater than the subgroup with T1/T2 at 12th month (95% vs 67%, *P* = 0.024) and 18th month (95% vs 67%, *P* = 0.024) in the MCALP group (Supplementary Table 5). Additionally, the complete remission percentages of patients without C1/C2 in the MCALP group was greater than the subgroup with C1/C2 at 18th month (67% vs 17%, *P* = 0.020). And, the total remission percentage in the subgroup without C1/C2 was greater than the subgroup with C1/C2 at 12th month (67% vs 95%, *P* = 0.024) and 18th month (67% vs 95%, *P* = 0.024) in the MCALP group (Supplementary Table 6). No patients in these two groups progressed to ESKD during the 12-month observational phase, although 4 patients in the MCALP group and 4 patients in the FP group did not relieve at the end of the trial.

### Kidney function changes in follow-up

**Figure 3:**
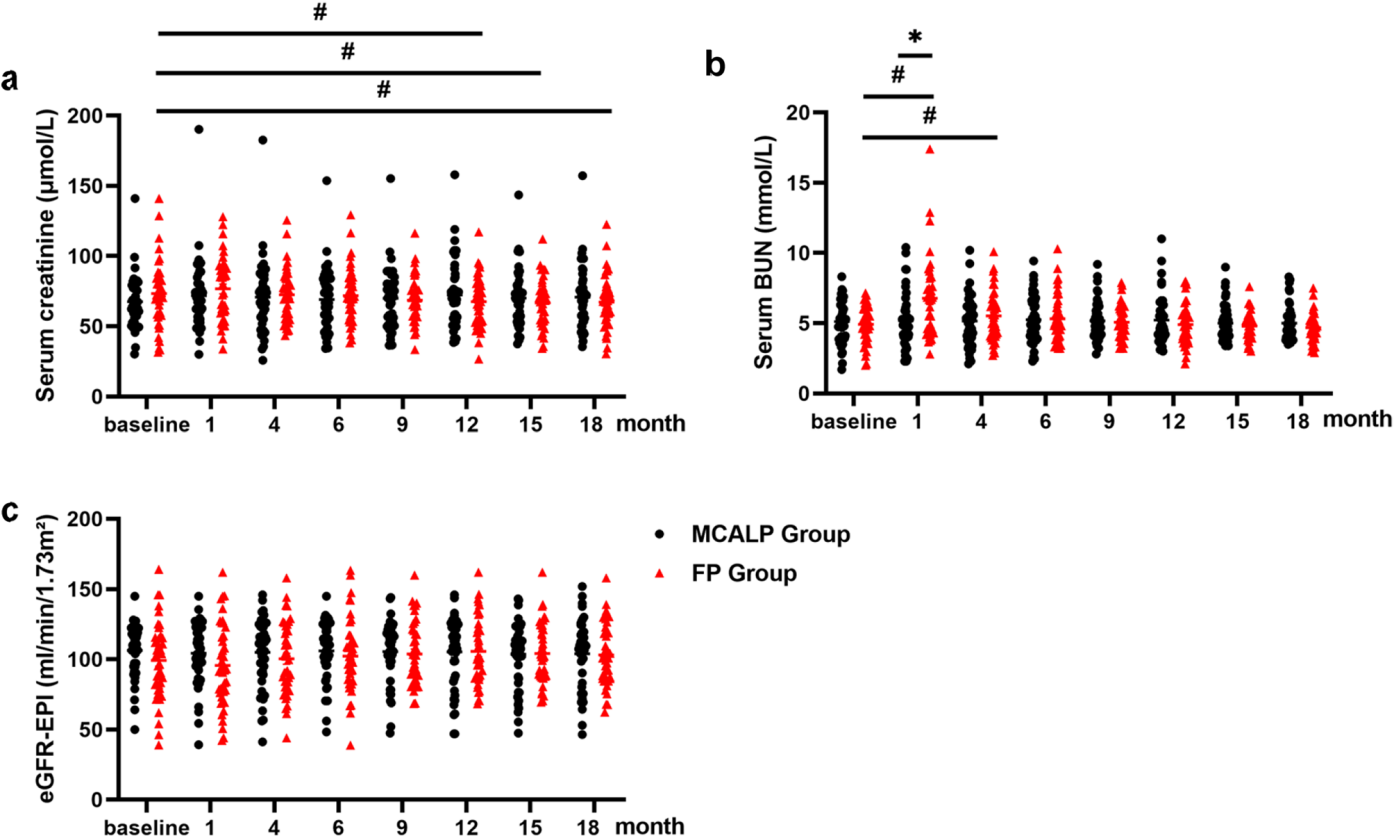
Absolute and relative change in serum BUN (**a**), serum creatinine (**b**), and eGFR-EPI (**c**), in two groups from baseline to 18 months. *P < 0.05 MCALP group *vs* FP group. ^#^*P* < 0.05 follow-up *vs* baseline respectively.

We next assessed the kidney function changes including serum BUN, serum creatinine levels, and eGFR-EPI in the MCALP and FP groups. In the MCALP group, serum BUN remained stable during the follow-up ((0.10 [− 0.26 to 0.47] vs 0.05 [− 0.29 to 0.40] mmol/L, at 12th month, *P* = 0.373) (− 0.12 [− 0.48 to 0.25] vs − 0.17 [− 0.53 to 0.19] mmol/L, at 18th month, *P* = 0.206)) (Fig. 3a). Compared to baseline, serum BUN levels in the FP group were significantly increased at 1st month (1.68 [1.40 to 1.97] mmol/L, *P* = 0.001) and 4th month (0.57 [0.37 to 0.77] mmol/L, *P* < 0.001). It may be explained that patients had a high catabolism state due to taking the full dose of prednisone, such as abnormal carbohydrate and protein metabolism, which led to an increase in serum BUN, this kind of change in BUN gradually disappeared after reducing the dosage of prednisone^22^. In MCALP group, there were no significant difference in the serum BUN levels at the 1st month (0.20 [− 0.04 to 0.44] mmol/L, *P* = 0.347) and at 4th month (0.31 [0.12 to 0.49] mmol/L, *P* = 0.658) compared with that in baseline. There was no difference in the serum BUN levels between the two groups at baseline, 6th, 9th,12th,15th, and 18th month.

Changes in serum creatinine and eGFR-EPI from baseline to 18 months in two groups were shown in Fig. 3b,c. In the FP group, serum creatinine was significantly decreased at the 12th month (− 3.35 [− 5.16 to − 1.53] μmol/L, *P* = 0.001), 15th month (− 5.09 [− 5.73 to − 4.45] μmol/L, *P* < 0.001) and the 18th month (− 6.33 [− 6.97 to − 5.69] μmol/L, *P* < 0.001). No significant difference was detected on the eGFR-EPI between baseline and 1st, 4th, 6th, 9th,12th, 15th, 18th month during the observational phase in either group respectively, neither significant changes between the two groups as well.

### Blood pressure and metabolic profiles change in follow-up

Next, we assessed the blood pressure and metabolic profiles including body weight, total cholesterol levels, triglyceride levels and fasting blood glucose levels in MCALP and FP groups. There was no difference in the blood pressure between these groups at baseline, 4th, 6th, 12th, and 18th month shown in Supplementary Fig. 1a,b. Compared to baseline, systolic blood pressure levels in the MCALP group were decreased at 4th month (− 1.82 [− 3.80 to 0.15] mmHg, *P* = 0.070), 12th month (− 5.56 [− 8.38 to − 2.73] mmHg, *P* < 0.001) and 18th month (− 7.91 [− 13.39 to − 2.43] mmHg, *P* = 0.006), while diastolic blood pressure levels were decreased at 12th month (− 1.82 [− 3.80 to − 0.15] mmHg, *P* = 0.070) and 18th month (− 1.82 [− 3.80 to − 0.15] mmHg, *P* = 0.006). Meanwhile, systolic blood pressure levels in the FP group remained stable during the follow-up ((0.88 [− 0.76 to 2.52] mmHg at 4th month, *P* = 0.285), (0.45 [− 1.12 to 2.03] mmHg at 6th month, *P* = 0.565), (− 1.55 [− 3.75 to 0.66] mmHg at 12th month, *P* = 0.165), (− 1.93 [− 4.25 to 0.39] mmHg at 18th month, *P* = 0.101)). However, diastolic blood pressure levels in FP group were significantly decreased at 12th month (2.57 [0.24 to 4.90] mmHg, *P* = 0.031).

Changes in body weight from baseline to 18 months in two groups were shown in Supplementary Fig. 1c. There was no difference in the body weight between the two groups at baseline, 4th, 6th, 12th, and 18th month. Compared to baseline, body weight levels in the MCALP and FP group were significantly increased at 4th month (0.60 [0.33 to 0.87] kg, *P* < 0.001 vs 1.56 [0.99 to 2.13] kg, *P* < 0.001), 6th month (0.82 [0.42 to 1.22] kg, *P* < 0.001 vs 0.92 [0.40 to 1.43] kg, *P* = 0.001), 12th month (0.82 [0.41 to 1.23] kg, *P* < 0.001 vs 0.67 [0.15 to 1.18] kg, *P* = 0.012) and 18th month (0.69 [0.26 to 1.11] kg, *P* = 0.002 vs 0.61 [0.09 to 1.12] kg, *P* = 0.022) respectively.

Changes in total cholesterol and triglyceride from baseline to 18 months in two groups were shown in Supplementary Fig. 1d,e. There was no difference in the total cholesterol levels and triglyceride levels between the two groups at baseline, 4th, 6th, 12th, and 18th month respectively. In the MCALP group, compared to baseline, total cholesterol levels were significantly increased at 4th month(0.66 [0.32 to 1.01] mmol/L, *P* < 0.001) and 6th month (0.31 [0.03 to 0.59] mmol/L, *P* = 0.030). Meanwhile, compared to baseline, triglyceride levels were significantly increased at 4th month (0.44 [0.18 to 0.72] mmol/L, *P* = 0.002). Compared to baseline, total cholesterol levels were significantly increased at 4th month (0.79 [0.38 to 1.20] mmol/L, *P* < 0.001), and triglyceride levels were significantly increased at 4th month (0.44 [0.11 to 0.78] mmol/L, *P* = 0.011) and 6th month(0.25 [0.01 to 0.50] mmol/L, *P* = 0.045) in the FP group.

Changes in fasting blood glucose levels from baseline to 18 months in two groups were shown in Supplementary Fig. 1f. At the 4th month, fasting blood glucose levels in the MCALP group were greater than that in the FP group (5.07 ± 0.73 mmol/L vs 4.78 ± 0.57 mmol/L, *P* = 0.043). In the MCALP group, fasting blood glucose levels were significantly increased at 4th month (0.33 [0.12 to 0.54] mmol/L, *P* = 0.003), 6th month (0.32 [0.15 to 0.49] mmol/L, *P* < 0.001), 12th month (0.33 [0.15 to 0.52] mmol/L, *P* = 0.001) and 18th month (0.31 [0.13 to 0.48] mmol/L, *P* = 0.001). And it remained stable in the FP group during the follow-up observational phase. The slightly higher baseline BMI in the MCALP group patients might in part contribute to the change discrepancy in the fasting blood glucose between these two groups, that likely to increase fasting blood glucose levels^23^. The pulsed methylprednisolone used might cause a loss of pancreatic adaptive response due to an acute and supra-physiological steroid load^24^, it also might cause different types of beta-cell dysfunction^23,25,26^. The fasting blood glucose levels in these two groups of patients were within the normal range.

### Safety and adverse events in follow-up

We next assessed the adverse events that happened in MCALP and FP groups during 18-month periods. Incidences of infections, particularly urinary tract infection (3% vs 10%, *P* = 0.025), weight gain (4% vs 19%, *P* = 0.033) and Cushing syndrome (3% vs 18%, *P* = 0.001 ) were significantly lower in the MCALP group than that in the FP group (Table 3). Patients with infection had improvement after receiving appropriate medication. Patients with impaired glucose tolerance and weight gain got improvement after diet control. Patients with hip discomfort were excluded from femoral head necrosis confirmed by X-ray and MRI examination. None of the death, osteonecrosis, fracture, cataract and ocular hypertension occurred in either treatment group during the observational phase.

**Table 3:** Adverse events occur in groups within 18 months.

Variables	MCALP group (N = 45)	FP group (N = 42)	*P* value
Total adverse events	28 (62)	30 (71)	0.363
**Infections**^**a**^	8 (18)	21 (50)	0.001
Pneumonia	1 (2)	3 (7)	0.273
Upper respiratory tract infection	5 (11)	9 (21)	0.191
Urinary tract infection	3 (7)	10 (24)	0.025
Weight gain	2 (4)	8 (19)	0.033
Insomnia	6 (13)	8 (19)	0.469
eGFR-EPI decline ≥ 20% from baseline	4 (9)	7 (17)	0.275
Impaired glucose tolerance	7 (16)	3 (7)	0.234
Newly diagnosed diabetes	0 (0)	1 (2)	0.298
Hyperuricemia	9 (20)	7 (17)	0.688
Hyperlipidemia	22 (49)	29 (69)	0.056
Cushing syndrome	3 (7)	18 (43)	0.001
Gastrointestinal symptoms	0 (0)	1 (2)	0.298
Hip discomfort	0 (0)	2 (5)	0.139

Values are given as number (percentage) and the number represents the number of events.

^a^Multiple occurrences of the same event(infections) in 1 person were only counted once. *P* value for comparisons between the number of patients in MCALP group and FP group.

## Discussion

IgAN, as a common primary glomerular disease in Asia, Europe, and the United States of America, has a heterogeneous clinical manifestation, which is still lacking safe and effective treatment. Some IgAN patients present few or no clinical manifestations for many years and most IgAN patients have been not diagnosed until progressing to chronic kidney disease (CKD) with developing many complications such as hypertension, anemia, or even reach renal failure due to their late presentation in hospitals^27^. Massive and persistent proteinuria is one of the independent risk factors for the disease progression of IgAN patients to ESKD^7^. Rapidly decreasing proteinuria and keeping proteinuria at a lower level (< 0.5 g/24 h) is crucial for maintaining a stable kidney function. Until now, immunosuppressive therapy, mainly glucocorticoids, is recommended for patients with persistent proteinuria greater than 1 g/24 h despite optimized and tolerated angiotensin-converting enzyme inhibitors (ACEIs) or angiotensin AT(1)-receptor blockers (ARBs) administration including the KDIGO 2021 guidelines for the management of glomerular diseases regarding the IgAN treatment^10–12,14–16,28^. Despite the antiproteinuric efficacy of glucocorticoids in high-risk IgAN patients, the risk of serious adverse events from systemic glucocorticoid administration is up to 12–14% which is strongly correlated with dosage and duration of glucocorticoids utilization^29^. It is very challenging to address what dosage of glucocorticoid should be effectively and safely used in high-risk IgAN patients and how to use it? Oral administration or intravenous administration or both or in a sequential combination? Hence, the investigation of new treatment strategies for high-risk IgAN patients in the real world is necessary.

KDIGO 2012 guidelines for the management of glomerular diseases did not provide a recommendation regarding the treatment strategy of how to use the immunosuppressant for high-risk IgAN patients with proteinuria 1–3.5 g/day^30^, even in the newly released KDIGO 2021 guidelines for the management of glomerular diseases^10,11^. To address this question, we designed and conducted this prospective longitudinal cohort study clinical trial focusing on the high-risk IgAN patients with proteinuria ≤ 3.5 g/day. We compared the efficacy and safety who received pulsed intravenous methylprednisolone (0.5 g of methylprednisolone intravenously for three days, twice repeatedly during 6 months) combined with low-dose prednisone (oral prednisone at a dose of 15 mg every other day) with matched controls who received the full-dose prednisone therapy, and we observed a quick decrease of proteinuria in the first month and continued to drop until the end of the trial in both groups. More than 90% of patients responded to glucocorticoid, and there was no significant difference in complete remission rate between the two groups. The remission rate was higher than the findings reported by Pozzi et al.^14,16^. In their study of methylprednisolone therapy, approximately 30% of treated IgAN patients did not respond to glucocorticoid. But only less than 10% of patients in our study failed to respond to glucocorticoid treatment, it may be attributed to the difference in race and genetic background^31^.In addition, a retrospective study reported by Laranjinha et al.^17^ using a higher dose of glucocorticoid for treatment of 36 cases showed the findings that were consistent with our study. Our study suggested that pulsed intravenous methylprednisolone combined with alternative low-dose prednisone quickly and effectively reduced proteinuria and might be no inferior to that of full-dose prednisone treatment in high-risk IgAN patients during the 18-month study phase. There is no difference in the rate of relapse between the two groups, which is different from the aforementioned study, probably because of our shorter follow-up time of 18 months. All patients in our study received comprehensive supportive care for 90 days before enrolling in the trial and maintained SBP < 120 mmHg which was in line with the requirement of KDIGO guidelines^10,11^.

Although the KDIGO 2021 guidelines for the management of glomerular diseases affirmed the importance of the Oxford classification of MEST-C scores in IgAN management, no treatment plan is recommended according to the classification, except that IgAN patients characterized with rapidly progressive glomerulonephritis (RPGN) and/or glomerular crescent formation in the kidney biopsy tissue were recommended to follow anti-neutrophil cytoplasmic antibody-related vasculitis (AAV) management. In the recently reported prospective clinical trials, neither the STOP-IgAN study^28^ nor the TESTING study^12^ or the NEFIGAN study^13^ analyzed the possible correlation between renal pathological lesions scored by the MEST-C classification with treatment, prognosis in IgAN patients. A retrospective study (VALIGA) indicated more favorable improvement of kidney function decline using glucocorticoids in those IgAN patients characterized with M1, E1, S1, and T1-2 compared to that of patients with M0, E0, S0, and T0^32^. In our study, the percentages of complete remission were similar in the subgroup with E1, S1, or C1/C2 between these two groups. However, there were higher percentages of complete remission in the subgroup without E1, S1, or C1/C2 compared to the subgroup with E1, S1, or C1/C2 in the MCALP group at the 6th month upon completion of treatment, and the tendency continued to 12th month and 18th month. These findings on the percentage of complete remission between the subgroup without E1, S1, or C1/C2 with the subgroup with E1, S1, or C1/C2 in the FP group were not observed, which may be limited by insufficient patient cases in the subgroup with E1 (6 patients) or with C1/C2 (3 patients). This result suggests that pulsed intravenous methylprednisolone combined with alternative low-dose prednisone might have potentially better efficacy in patients without E1, S1, or C1/C2 on the kidney pathological findings, which is consistent with the previous studies^32^. However, the relationship between the Oxford MEST-C kidney pathology classification and the initiation of appropriate immunosuppressant treatment in IgAN patients needs more studies and evidence.

Furthermore, the subgroup without T1/T2 in the MCALP group showed the higher total remission rate at 12th and 18th months compared to the subgroup with T1/T2, but the complete remission percentage and partial remission percentage did not exhibit the statistical difference between the two subgroups. The reason for this might be that the total patient cases in the subgroup with T1/T2 is only 6 and the follow-up time is too short in this trial. The MEST-C scores in the MCALP group seem to be more severe than those in the FP group, although there was no statistical difference. Therefore, although there is no significant difference in the percentage of the total remission between the two groups according to the MEST-C subgroup analysis, it can be seen that the IgAN treatment strategies need to be further adjusted according to renal pathological findings, which indicates that when E1, S1, T1/2 or C1/C2 present, glucocorticoid might have potential benefit, especially pulsed steroid therapy.

In the study, serum BUN levels of patients in the FP group increased suddenly at the 1st month after treatment, and it was higher than in the MCALP group. Meanwhile, serum albumin levels increased significantly after intravenous methylprednisolone compared with full-dose prednisone treatment. As such, we speculate that an increase of serum BUN levels in the FP group might be associated with an improvement of plasma protein levels. The findings have not been mentioned in previously reported studies. Pozzi et al.^14^ found that glucocorticoid therapy was beneficial to protect the kidney function of IgAN patients and delayed the disease progression after a follow-up of 5–10 years. We found proteinuria was under a lower level consistently during the observational phase of 12 months after 6-month treatment. The long-term antiproteinuric efficacy and protection of kidney function in these patients between the MCALP and the FP therapy are under further follow-up.

The most common side-effects of systemic glucocorticoid treatment are Cushing syndrome, weight gain and infections. Most adverse events occurred in the first 3 months after glucocorticoid treatment, and about half of the adverse events were reported in the first 6 months of follow-up^12,29^. In the study, there was a lower rate of adverse events in the MCALP group in which the dosage of prednisone was 2/3 of the FP group and less than 1/2 of the dosage used in Pozzi’s study^16^. None of both groups of patients encountered serious adverse events during the 18-month study. One patient in the FP group was diagnosed with diabetes at the end of the trial, only seven patients suffered from impaired glucose tolerance and nobody had been diagnosed with diabetes in the MCALP group. The STOP-IgA study showed that glucocorticoid could decrease proteinuria but side effects were higher compared with the supportive care group^20,21^. We thought that the dosage of glucocorticoid in the STOP-IgA study was higher than that in our study and the eGFR of enrolled patients was lower which could make patients be susceptible to kinds of infections. In the TESTING study^12^, the results also showed that methylprednisolone treatment for 6–8 months could significantly alleviate proteinuria and prolonged the decline of eGFR, but adverse events were serious. Hence, the investigators changed their scheme to decrease the dosage of methylprednisolone as 0.4–0.6 mg/kg/day in the ongoing TESTING Low Dose study (NCT01560052). The total dosage of glucocorticoid in our study was similar to the TESTING Low Dose study.

However, this study still has some limitations: single-center enrolled Asian patients; a short follow-up period; insufficient patient cases for some subgroups of Oxford MEST-C kidney pathology classification. Additionally, a specific podocytopathic variant of IgAN or the existence of features of minimal change disease (MCD) with diffuse podocyte foot process effacement in IgAN patients were recently reported^33,34^, steroid therapy was indicated an appropriate therapeutic strategy in those patients, which was also recommended by the newly-released KDIGO 2021 guidelines for the management of glomerular diseases^10,11^. A multicenter, double-blind, broader inclusion criteria and long-term follow-up trial are needed in the future.

In conclusion, this study suggests that the efficacy of reduction of proteinuria and protection of kidney function is similar between the pulsed intravenous methylprednisolone combined with low-dose prednisone and full-dose prednisone therapy over 18 months in high-risk IgAN patients. However, pulsed intravenous methylprednisolone combined with low-dose prednisone treatment was safer than the full-dose prednisone treatment. Therefore, the treatment of pulsed intravenous methylprednisolone combined with low-dose prednisone is a possible new treatment option for high-risk IgAN patients to the nephrologists in their clinical practice.

## Materials and methods

### Statement of ethics

This study was approved by the by the Medical Ethics Committee of The Second Affiliated Hospital of Xi’an Jiaotong University, and the approval number was No. [2021] 008. The study was also approved by the Chinese Clinical Trial Registry (registration date 13/01/2018, approval number ChiCTR1800014442, https://www.chictr.org.cn/), and all participants in this study provided written informed consent. The study was carried out in accordance with the Declaration of Helsinki and the principles outlined in the declaration.

### Study design and population

**Figure 4:**
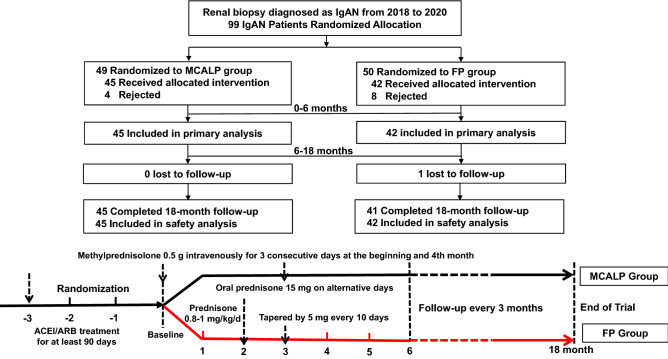
Study recruitment (the inclusion workflow of the study). The primary analysis sets and the safety analysis sets excluded patients who did not receive the allocated intervention. MCALP, methylprednisolone combined with alternative low-dose prednisone; FP, full-dose prednisone.

We conducted a prospective, open-label, randomized, controlled, 18-month trial with a two-group, parallel, group-sequential design. The study enrolled 87 renal biopsy-proven IgAN Chinese patients after screening 268 IgAN cases in the Department of Nephrology at the Second Affiliated Hospital of Xi'an Jiaotong University from February 5, 2018, to January 20, 2020. The inclusion workflow of the study was shown in Fig. 4. One patient in the FP group was lost to follow-up because this patient could not be contacted successfully. Inclusion criteria were as follows: (1) diagnosed with IgAN by renal biopsy; (2) age of 15 to 75 years; (3) urinary protein excretion ≥ 1.0 g/24 h and ≤ 3.5 g/24 h, stable blood pressure after comprehensive supportive care (including ACEI or ARB therapy receiving the maximum labeled or tolerated dose according to guidelines of KDIGO for IgAN management) for at least 90 days; (4) serum creatinine < 2.0 mg/dL (171 μmol/L) and eGFR-EPI > 30 ml/min/1.73 m^2^. In both groups, patients were allowed to take antihypertensive drugs, diuretics, and antiplatelet aggregation drugs when needed. The exclusion criteria included the IgAN patients from a secondary cause; usage of glucocorticoids or immunosuppressive therapy within the three previous years; active gastrointestinal ulcer; viral hepatitis; serious life-threatening infections; other autoimmune diseases; severe heart and lung dysfunction; pregnancy or lactation.

A randomization list was scientifically created by a random number table coming from the appendix of the Chinese textbook named Medical Statistics (Version 2) with block randomization of 4 subjects as a group. Patients were randomly assigned to the MCALP group or FP group in a 1:1 ratio.

Glucocorticoid treatment was initiated when the patients presented with proteinuria ≥ 1.0 g/24 h and ≤ 3.5 g/24 h after optimized supportive treatment for more than 90 days. In the MCALP group, IgAN patients received 0.5 g of methylprednisolone intravenously for three consecutive days at baseline, 3th month, followed by oral prednisone at a dose of 15 mg every other day for 6 months. In the FP group, 0.8–1.0 mg/kg/day of prednisone (maximum 70 mg/day) was given to them at the beginning of the study, then tapered by 5 mg every 10 days for the next 4 months (Fig. 4). Two groups are both followed up 12-month. Hypertension was defined as blood pressure ≥ 140/90 mmHg. The IgAN patients who used other immunosuppressants like leflunomide, cyclosporine A, cyclophosphamide, and methotrexate were not enrolled. All drugs were administered as part of general medical care and were not donated specifically for the study.

### Data collection and outcome measures

The data for age, gender, blood pressure, body weight, and many laboratory indicators (such as albumin, serum creatinine levels) at baseline in all patients were collected. Patients were followed up regularly at months 1, 4, 6, 9, 12, 15 and 18. At each follow-up, the blood pressure, body weight, 24 h urinary protein excretion levels, serum albumin levels, blood urea nitrogen (serum BUN) levels, serum creatinine levels, eGFR calculated by the CKD-EPI formula, blood routine testing, blood lipids, and fasting blood glucose were assessed and recorded. The adverse events were recorded as well. The score of kidney biopsy was based on Lee's grading system^35^ and the Oxford Classification of IgAN (MEST-C score)^36^. M1 was defined as a mesangial hypercellularity score > 0.5, E1 was defined as the presence of endocapillary hypercellularity, S1 was defined as the presence of segmental glomerulosclerosis, T1 was defined as tubular atrophy/interstitial fibrosis within 26–50% and T2 was defined as tubular atrophy/interstitial fibrosis > 50% of the kidney cortical area. C1 was defined as cellular/fibrocellular crescent present in at least 1 glomerulus, and C2 was defined as cellular/fibrocellular crescent present in > 25% of glomeruli.

The primary endpoint was the complete remission rate at the 6th,12th, and 18th months. Complete remission was defined as proteinuria ≤ 0.3 g/24 h with a normal serum creatinine level. Partial remission was defined as proteinuria > 0.3 g/24 h but > 50% decline from baseline, serum albumin level ≥ 35 g/L. No response was defined as not meeting the criteria of complete remission or partial remission or an increase in serum creatinine level more than doubling baseline or a 50% decrease in eGFR-EPI from baseline^37^. Total remission rate was expressed as the rate of complete remission plus the rate of partial remission. Relapse was defined as complete or partial remission followed by proteinuria > 1.0 g/24 h on 2 consecutive measurements. Weight gain was defined as a weight gain > 3 kg on 3 consecutive measurements. The severe adverse events were defined as follows: death; a life-threatening situation requiring inpatient hospitalization or prolongation of existing hospitalization, or serious infections, or gastrointestinal hemorrhage, or new-onset diabetes mellitus, or new-onset cataract, or severe organ failure events, or significant disability^38^.

### Statistical analyses

Statistical analyses were performed by using SPSS software (IBM SPSS statistics 20). The data of normal distribution or approximately normal distribution were represented by the mean ± standard deviation (SD). Median with interquartile (IQR) was used if data were not continuous variables. The categorical data were summarized as counts and percentages. The *t* test, Chi-square test, and Mann–Whitney U test were used for comparison between two groups as appropriate. The correlation analysis was performed by using Spearman rank correlation analysis. A *P* value less than 0.05 was considered statistically significant.

## Supplementary Information

Supplementary Information.
