# Supplementary material for: Effect of pulsed intravenous methylprednisolone with alternative low-dose prednisone on high-risk IgA nephropathy: a 18-month prospective clinical trial
Source: Sci Rep. 2022 Jan 7;12:255. doi: 10.1038/s41598-021-03691-0 (PMC8742122; doi:10.1038/s41598-021-03691-0)

Effect of Pulsed Intravenous Methylprednisolone with Alternative Low-dose Prednisone on High-Risk IgA Nephropathy: A 18-Month Prospective Clinical Trial

Yan Li[a,1], Rongguo Fu[a,1], Jie Gao[a], Li Wang[a], Zhaoyang Duan[a], Lifang Tian[a], Heng Ge[a], Xiaotao Ma[a], Yuzhan Zhang[a], Ke Li[a], Peihao Xu[b], Xuefei Tian[c,*], Zhao Chen[a,**]

[a]Department of Nephrology, The Second Affiliated Hospital of Xi'an Jiaotong University, Xi'an, Shaanxi, 710004, China

[b]School of Medicine, Royal College of Surgeons in Ireland, 123 St Stephen's Green, Dublin 2, Ireland

[c]Section of Nephrology, Department of Internal Medicine, Yale University School of Medicine, New Haven, Connecticut, 06520, USA.

[1]contributed equally to this work.

**Corresponding Author: Zhao Chen,
Department of Nephrology,
The Second Affiliated Hospital of Xi'an Jiaotong University,
157 West Fifth Road, Xi'an, Shaanxi 710004, China
E-mail: icetsblueluya@126.com.
*Co-Corresponding Author:Xuefei Tian
Section of Nephrology,
Department of Internal Medicine,
Yale University School of Medicine, New Haven, Connecticut, 06520, USA.
E-mail: xuefei.tian@yale.edu

The study approved by the Chinese Clinical Trial Registry (registration date 13/01/2018, approval number ChiCTR1800014442, https://www.chictr.org.cn/)

Supplementary table 1: The rate of complete remission at 1st , 4th , 6th , 9th ,12th ,15th and 18th month. Abbreviations: CI, confidence interval. n/N means event numbers/total numbers.

**Variables**	**MCALP Group**		**FP Group**		
	**n/N**	**Percentage (95%CI)**	**n/N**	**Percentage (95%CI)**	***P* value**
**1st month**	7/45	16%(5% to 27%)	6/42	14%(3% to 25%)	0.868
**4th month**	13/45	29%(15% to 43%)	11/42	26%(12% to 40%)	0.778
**6th month**	18/45	40%(25% to 55%)	23/42	55%(39% to 71%)	0.168
**9th month**	20/45	44%(29% to 60%)	23/41	56%(40% to 72%)	0.280
**12th month**	23/45	51%(36% to 66%)	24/41	58%(43% to 74%)	0.490
**15th month**	25/45	56%(41% to 71%)	23/41	56%(40% to 72%)	0.960
**18th month**	27/45	60%(45% to 75%)	23/41	56%(40% to 72%)	0.714

Supplementary table 2: The rate of partial remission，complete remission and total remission at 6th,12th and 18th month in subgroups with M1 and without M1. Abbreviations: CI, confidence interval. n/N means event numbers/total numbers. P^a, compared the difference between subgroup with M1 and without M1 in MCALP and FP group. P^b, compared the difference between MCALP and FP group in subgroup with M1. P^c , compared the difference between MCALP and FP group in subgroup without M1.

**Variables**	**MCALP Group**					**FP Group**					***P^b* value**	***P^c* value**
	**With M1**		**Without M1**		***P^a* value**	**With M1**		**Without M1**		***P^a* value**		
	**n/N**	**Percentage(95%CI)**	**n/N**	**Percentage(95%CI)**		**n/N**	**Percentage(95%CI)**	**n/N**	**Percentage(95%CI)**			
**Month 6**												
**Partial remission**	17/31	55%(36% to 73%)	6/14	43%(13% to 73%)	0.457	10/26	38%(18% to 59%)	5/16	31%(6% to 57%)	0.636	0.200	0.886
**Complete remission**	11/31	36%(18% to 53%)	7/14	50%(20% to 80%)	0.357	14/26	54%(33% to 74%)	9/16	56%(29% to 84%)	0.879		
**Total remission**	28/31	90%(80% to 100%)	13/14	93%(77% to 100%)	0.782	24/26	92%(81% to 100%)	14/16	88%(69% to 100%)	0.606		
**Month 12**												
**Partial remission**	15/31	48%(30% to 67%)	3/14	21%(0% to 46%)	0.087	9/26	35%(15% to 54%)	4/15	27%(1% to 52%)	0.598	0.701	0.847
**Complete remission**	14/31	45%(27% to 64%)	9/14	64%(36% to 93%)	0.235	14/26	54%(33% to 74%)	10/15	67%(40% to 94%)	0.422		
**Total remission**	29/31	94%(84% to 100%)	12/14	86%(65% to 100%)	0.393	23/26	88%(75% to 100%)	14/15	93%(79% to 100%)	0.613		
**Month 18**												
**Partial remission**	13/31	42%(24% to 60%)	1/14	7%(0% to 23%)	0.020	10/26	39%(18% to 59%)	4/15	27%(1% to 52%)	0.443	0.775	0.715
**Complete remission**	16/31	52%(33% to 70%)	11/14	79%(54% to 100%)	0.087	13/26	50%(29% to 71%)	10/15	67%(40% to 94%)	0.300		
**Total remission**	29/31	94%(84% to 100%)	12/14	86%(65% to 100%)	0.393	23/26	88%(75% to 100%)	14/15	93%(79% to 100%)	0.613		

Supplementary table 3: The rate of partial remission， complete remission and total remission at 6th,12th and 18th month in subgroups with E1 and without E1.
Abbreviations: CI, confidence interval. n/N means event numbers/total numbers. P^a, compared the difference between subgroup with E1 and without E1 in MCALP and FP group. P^b, compared the difference between MCALP and FP group in subgroup with E1. P^c , compared the difference between MCALP and FP group in subgroup without E1.

Variables	MCALP Group					FP Group					P^b value	P^c value
	With E1		Without E1		P^a value	With E1		Without E1		P^a value		
	n/N	Percentage(95%CI)	n/N	Percentage(95%CI)		n/N	Percentage(95%CI)	n/N	Percentage(95%CI)			
**Month 6**												
**Partial remission**	9/12	75%(46% to 100%)	14/33	42%(25% to 60%)	0.053	4/6	67%(12% to 100%)	11/36	31%(15% to 46%)	0.087	0.820	0.339
**Complete remission**	2/12	17%(0% to 41%)	16/33	48%(31% to 66%)	0.054	1/6	17%(0% to 59%)	22/36	61%(44% to 78%)	0.043		
**Total remission**	11/12	92%(73% to 100%)	30/33	91%(81% to 100%)	0.937	5/6	83%(40% to 100%)	33/36	92%(82% to 100%)	0.520		
**Month 12**												
**Partial remission**	9/12	75%(46% to 100%)	9/33	27%(11% to 43%)	0.004	3/6	50%(0% to 100%)	10/35	29%(13% to 44%)	0.297	0.437	0.983
**Complete remission**	3/12	25%(0% to 54%)	20/33	61%(43% to 78%)	0.035	3/6	50%(0% to 100%)	21/35	60%(43% to 77%)	0.646		
**Total remission**	12/12	100%	29/33	88%(76% to 100%)	0.206	6/6	100%	31/35	89%(77% to 100%)	0.383		
**Month 18**												
**Partial remission**	8/12	67%(35% to 98%)	6/33	18%(4% to 32%)	0.002	4/6	67%(13% to 100%)	10/35	29%(13% to 44%)	0.069	1.000	0.486
**Complete remission**	4/12	33%(2% to 65%)	23/33	70%(53% to 86%)	0.028	2/6	33%(0% to 88%)	21/35	60%(43% to 77%)	0.224		
**Total remission**	12/12	100%	29/33	88%(76% to 100%)	0.206	6/6	100%	31/35	89%(77% to 100%)	0.383		

Supplementary table 4: The rate of partial remission，complete remission and total remission at 6th,12th and 18th month in subgroups with S1 and without S1.
Abbreviations: CI, confidence interval. n/N means event numbers/total numbers. P^a, compared the difference between subgroup with S1 and without S1 in MCALP and FP group. P^b, compared the difference between MCALP and FP group in subgroup with S1. P^c , compared the difference between MCALP and FP group in subgroup without S1.

Variables	MCALP Group					FP Group					P^b value	P^c value
	With S1		Without S1		P^a value	With S1		Without S1		P^a value		
	n/N	Percentage(95%CI)	n/N	Percentage(95%CI)		n/N	Percentage(95%CI)	n/N	Percentage(95%CI)			
**Month 6**												
**Partial remission**	21/36	58%(41% to 75%)	2/9	22%(0% to 56%)	0.053	11/29	38%(19% to 57%)	4/13	31%(2% to 60%)	0.654	0.443	0.845
**Complete remission**	12/36	33%(17% to 49%)	6/9	67%(28% to 100%)	0.068	14/29	48%(29% to 68%)	9/13	69%(40% to 98%)	0.207		
**Total remission**	33/36	92%(82% to 100%)	8/9	89%(63% to 100%)	0.793	25/29	86%(73% to 100%)	13/13	100%	0.159		
**Month 12**												
**Partial remission**	16/36	44%(27% to 62%)	2/9	22%(0% to 56%)	0.224	10/28	36%(17% to 55%)	3/13	23%(0% to 50%)	0.418	0.812	1.000
**Complete remission**	16/36	44%(27% to 62%)	7/9	78%(44% to 100%)	0.074	14/28	50%(30% to 70%)	10/13	77%(50% to 100%)	0.103		
**Total remission**	32/36	89%(78% to 100%)	9/9	100%	0.295	24/28	86%(72% to 100%)	13/13	100%	0.151		
**Month 18**												
**Partial remission**	13/36	36%(20% to 53%)	1/9	11%(0% to 37%)	0.147	11/28	39%(20% to 59%)	3/13	23%(0% to 50%)	0.308	0.591	0.647
**Complete remission**	19/36	53%(36% to 70%)	8/9	89%(63% to 100%)	0.048	13/28	46%(27% to 66%)	10/13	77%(50% to 100%)	0.067		
**Total remission**	32/36	89%(78% to 100%)	9/9	100%	0.295	24/28	86%(72% to 100%)	13/13	100%	0.151		

Supplementary table 5: The rate of partial remission，complete remission and total remission at 6th,12th and 18th month in subgroups with T1/T2 and without T1/T2. Abbreviations: CI, confidence interval. n/N means event numbers/total numbers. P^a, compared the difference between subgroup with T1/T2 and without T1/T2 in MCALP and FP group. P^b, compared the difference between MCALP and FP group in subgroup with T1/T2 . P^c , compared the difference between MCALP and FP group in subgroup without T1/T2.

Variables	MCALP Group					FP Group					P^b value	P^c value
	With T1/T2		Without T1/T2		P^a value	With T1/T2		Without T1/T2		P^a value		
	n/N	Percentage(95%CI)	n/N	Percentage(95%CI)		n/N	Percentage(95%CI)	n/N	Percentage(95%CI)			
**Month 6**												
**Partial remission**	5/6	83%(40% to 100%)	18/39	46%(30% to 63%)	0.090	1/3	33%(0% to 100%)	14/38	37%(21% to 53%)	0.903	0.095	0.483
**Complete remission**	0/6	0%	18/39	46%(30% to 63%)	0.032	2/3	67%(0% to 100%)	21/38	55%(39% to 72%)	0.702		
**Total remission**	5/6	83%(40% to 100%)	36/39	92%(84% to 100%)	0.472	3/3	100%	35/38	92%(83% to 100%)	0.613		
**Month 12**												
**Partial remission**	3/6	50%(0% to 100%)	15/39	38%(22% to 54%)	0.591	1/3	33%(0% to 100%)	12/37	32%(17% to 48%)	0.974	0.167	0.891
**Complete remission**	1/6	17%(0% to 59%)	22/39	56%(40% to 73%)	0.070	2/3	67%(0% to 100%)	22/37	59%(43% to 76%)	0.806		
**Total remission**	4/6	67%(12% to 100%)	37/39	95%(88% to 100%)	0.024	3/3	100%	34/37	92%(83% to 100%)	0.608		
**Month 18**												
**Partial remission**	3/6	50%(0% to 100%)	11/39	28%(13% to 43%)	0.283	1/3	33%(0% to 100%)	13/37	35%(19% to 51%)	0.950	0.167	0.362
**Complete remission**	1/6	17%(0% to 59%)	26/39	67%(51% to 82%)	0.020	2/3	67%(0% to 100%)	21/37	57%(40% to 74%)	0.738		
**Total remission**	4/6	67%(12% to 100%)	37/39	95%(88% to 100%)	0.024	3/3	100%	34/37	92%(83% to 100%)	0.608		

Supplementary table 6: The rate of partial remission，complete remission and total remission at 6th,12th and 18th month in subgroups with C1/C2 and without C1/C2. Abbreviations: CI, confidence interval. n/N means event numbers/total numbers. P[a], compared the difference between subgroup with C1/C2 and without C1/C2 in MCALP and FP group. P[b], compared the difference between MCALP and FP group in subgroup with C1/C2. P[c] , compared the difference between MCALP and FP group in subgroup without C1/C2.

Variables	MCALP Group					FP Group					*P*[b] value	*P*[c] value
	With C1/C2		Without C1/C2		*P*[a] value	With C1/C2		Without C1/C2		*P*[a] value		
	n/N	Percentage(95%CI)	n/N	Percentage(95%CI)		n/N	Percentage(95%CI)	n/N	Percentage(95%CI)			
**Month 6**												
**Partial remission**	4/6	67%(13% to 100%)	19/39	49%(32% to 65%)	0.413	1/3	33%(0% to 100%)	14/39	36%(20% to 52%)	0.929	0.301	0.516
**Complete remission**	1/6	17%(0% to 60%)	17/39	44%(27% to 60%)	0.210	2/3	67%(0% to 100%)	21/39	54%(38% to 70%)	0.667		
**Total remission**	5/6	83%(40% to 100%)	36/39	95%(88% to 100%)	0.472	3/3	100%	35/39	88%(80% to 100%)	0.560		
**Month 12**												
**Partial remission**	3/6	50%(0% to 100%)	15/39	39%(23% to 54%)	0.591	1/3	33%(0% to 100%)	12/39	31%(16% to 46%)	0.926	0.269	0.445
**Complete remission**	1/6	17%(0% to 60%)	22/39	56%(40% to 73%)	0.070	2/3	67%(0% to 100%)	22/39	56%(40% to 73%)	0.729		
**Total remission**	4/6	67%(13% to 100%)	37/39	95%(88% to 100%)	0.024	3/3	100%	34/39	87%(76% to 98%)	0.509		
**Month 18**												
**Partial remission**	3/6	50%(0% to 100%)	11/39	28%(13% to 43%)	0.283	1/3	33%(0% to 100%)	13/39	33%(18% to 49%)	1.000	0.269	0.371
**Complete remission**	1/6	17%(0% to 60%)	26/39	67%(51% to 82%)	0.020	2/3	67%(0% to 100%)	21/39	54%(38% to 70%)	0.667		
**Total remission**	4/6	67%(13% to 100%)	37/39	95%(88% to 100%)	0.024	3/3	100%	34/39	87%(76% to 98%)	0.509		

Supplementary figure 1: The changes of blood pressure(BP) (6a and 6b), body weight (6c), total cholesterol (6d), triglyceride (6e) and fasting blood glucose levels(6f) change in follow-up. * $P < 0.05$ MCALP group *vs* FP group. # $P < 0.05$ follow-up *vs* baseline respectively.

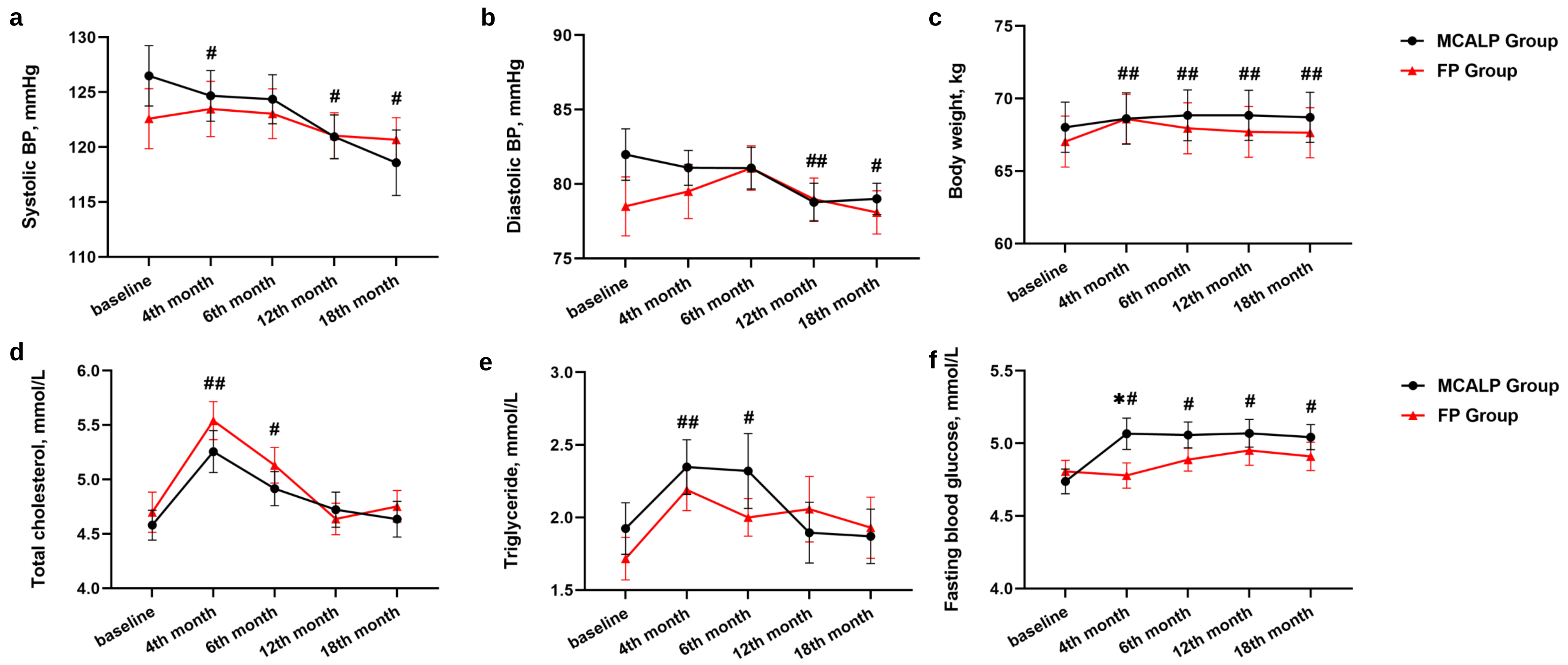

Supplement: Supplementary file 1 — Supplementary Information. [file 41598_2021_3691_MOESM1_ESM.pdf]
